# Supplementary material for: Stereotactic Body Radiotherapy for Patients with Lung Oligometastatic Disease: A Five-Year Systematic Review
Source: Cancers (Basel). 2021 Jul 20;13(14):3623. doi: 10.3390/cancers13143623 (PMC8303507; doi:10.3390/cancers13143623)
Supplement: Supplementary file 1 [file cancers-13-03623-s001.zip › cancers-1250825-supplementary.pdf]

cancers

MDPI

Supplementary Materials: Stereotactic Body Radiotherapy for Patients with Lung Oligometastatic Disease: a Five-Year Review

Guillaume Virbel, Clara Le Fèvre, Georges Noël and Delphine Antoni

Table S1. Study criteria of the 18 papers used for PRISMA method.

Author	Number of patients	Gender	Age	oligometastases definition	irradiated metastases	Synchronous or metachronous	Size of lesions	LC	OS	Toxicity	Total
Jung et al. 2015 [26]	d	ud	d	d	d	d	d	d	d	d	90%
Garcia-Cabezas et al. 2015 [18]	d	d	d	d	d	d	d	d	d	d	100%
Filippi et al. 2015 [20]	d	d	d	d	d	d	d	ud	d	d	90%
Navarria et al. 2015 [21]	d	d	d	d	d	d	d	d	d	d	100%
Wang et al. 2015 [29]	d	d	d	ud	d	ud	d	d	d	d	80%
Siva et al. 2015 [27]	d	d	d	d	d	ud	ud	d	d	d	80%
Lischalk et al. 2016 [31]	d	d	d	ud	d	ud	d	d	d	d	80%
Baumann et al. 2016 [30]	d	d	d	ud	d	ud	d	d	d	d	80%
Pasqualetti et al. 2017 [32]	d	d	d	ud	d	d	d	d	ud	d	80%
Agolli et al. 2017 [25]	d	d	d	d	d	d	d	d	d	d	100%
Lindsay et al. 2018 [33]	d	d	d	ud	d	ud	d	d	d	d	80%
Qiu et al. 2018 [16]	d	d	d	ud	d	ud	d	d	d	ud	70%

Osti et al. 2018 [17]	d	d	d	d	d	d	d	d	d	d	100%
Lee et al. 2018 [28]	d	d	d	d	d	ud	d	d	d	d	90%
Sharma et al. 2018 [23]	d	d	d	d	d	d	ud	d	d	d	90%
Li et al. 2019 [22]	d	d	d	d	d	d	d	d	d	d	100%
Helou et al. 2017 [19]	d	d	d	ud	d	ud	d	d	ud	d	70%
Berkovic et al. 2020 [24]	d	d	d	d	d	d	d	d	d	d	100%

d : defined ; LC : local control ; OS : overall survival ; ud : undefined.
